# A “Scientific” Estimate of Asphyxiation by Nitrous Oxide

**Published:** 1868-08

**Authors:** 


### A "SCIENTIFIC" ESTIMATE OF ASPHYXIATION BY NITROUS OXIDE.

A recent statement strenuously condemnatory of the use of this most deadly and invariably fatal agent for anæsthesia, of the famous Dr. Richardson, the renowned author of the ammonia theory of fibrine, challenges our highest admiration for the man, and establishes forever, on an impregnable basis, our previous confidence in him as a discoverer.

Dr. Richardson's statement is a denunciation of the use of nitrous oxide for anæsthesia, which he pronounces to be an undoubted asphyxiant.

This, as any one is perfectly aware who have used it frequently, is perfectly true.

Dr. Richardson, as every one knows, is a famous experimenter. The head, or at least front in rank, of that class of "scientific" men who find the result of an experiment always to be the veritable phenomena of nature herself;—not as furnishing the data for the reason, by means of which she may go on to discern the character of natural phenomena, —*i. e.* phenomena in the nature of things, which for a time elude our scrutiny,—but as the things and phenomena as naturally constituted themselves.

He has ascertained, by experiment, that bichloride of methylene is not only a perfectly harmless anæsthetic agent, but is in fact a *rather* more perfect respiratory element than common air,—being not only, in his estimation, as perfectly harmless as the latter, and exempt from its disabilities, but as conferring additional vitality on the human organism which inhales it. So that, on the whole, if it could be instituted for common air, and regularly breathed instead of it, and instead of, occasionally, as an anæsthetic, mankind would regain that immutability of the organism which was lost in the historic beginnings of the race.

To compare this strictly *recreative* substance, which at once gives us ease of our pains, and confers upon us a blissful and *wholesome* unconsciousness of the surgeon's skill in freeing the body from tormenting flesh and bone, with that strictly murderous agent, nitrous oxide, more deadly to inhalation than the poisonous effluence of the upas tree, is preposterous.

If, on the famous Doctor's reasons, the latter continues to be used to any greater extent, to that extent, *his* anæsthetic will not be, and hence, reasonably enough his peculiarly or rather *invariably* unenglish condemnation of nitrous oxide.

With a hardihood of candor, unmatched by man or (fallen) angel, with an infinite freedom from any personal consideration, with a boundless magnanimity and disinterestedness the Doctor denounces nitrous oxide as an unmixed evil, sure to asphyxiate every son of Adam who credulously inhales it, of no use to anybody but for the suicidal purposes of the despairing Frenchman, and as a substitute for the fumes of charcoal.

It is, indeed, one of the inexplicable anomalies of human society, that this agent of such greatly superior asphyxiating capacity, should never have been used in such cases for suicide instead of the troublesome and suffocating fumes of charcoal.

